# Supplementary material for: Oncolytic Activity of Targeted Picornaviruses Formulated as Synthetic Infectious RNA
Source: Mol Ther Oncolytics. 2020 May 19;17:484–95. doi: 10.1016/j.omto.2020.05.003 (PMC7276391; doi:10.1016/j.omto.2020.05.003)
Supplement: Document S1. Figures S1–S3 and Table S1 [file mmc1.pdf]

OMTO, Volume *17*

Supplemental Information

Oncolytic Activity of Targeted Picornaviruses Formulated as Synthetic Infectious RNA

Noura B. Elsedawy, Rebecca A. Nace, Stephen J. Russell, and Autumn J. Schulze

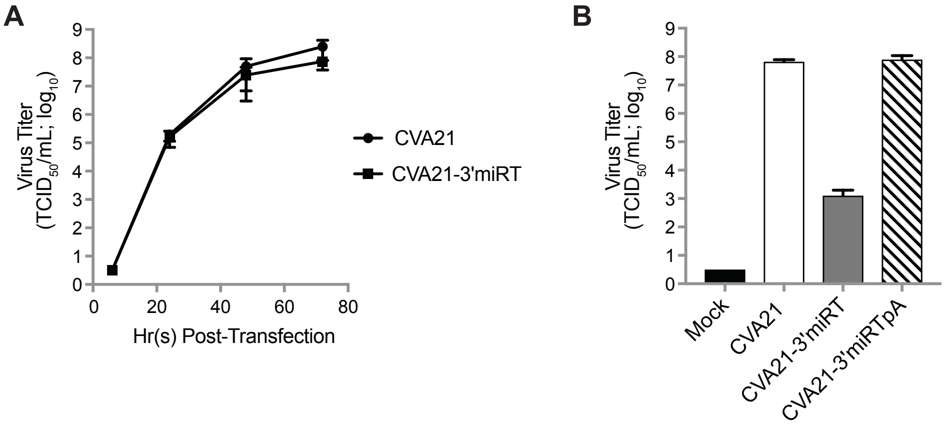

Figure S1. CVA21-3'miRT iRNA specific infectivity is temperature-sensitive and is improved with *in vitro* polyadenylation. (**A**) Infectious virus recovery time course in H1-HeLa cells at 32 °C. Cells were transfected with 1 µg of *in vitro*-derived RNA encoding CVA21 or CVA21-3'miRT genomes. Samples were collected at various times post transfection and infectious virus titrated. The experiment was run in quadruplicate and the data is represented as mean viral titers +/- standard error. (**B**) H1-HeLa cells were transfected with 1 µg of *in vitro*-derived RNA encoding CVA21, CVA21-3'miRT, or polyadenylated CVA21-3'miRT (pA). Recovered viruses were passaged twice in H1-HeLa cells and the cleared stocks titrated. The experiment was run in duplicate and that data are represented as mean viral titer +/- standard deviation.

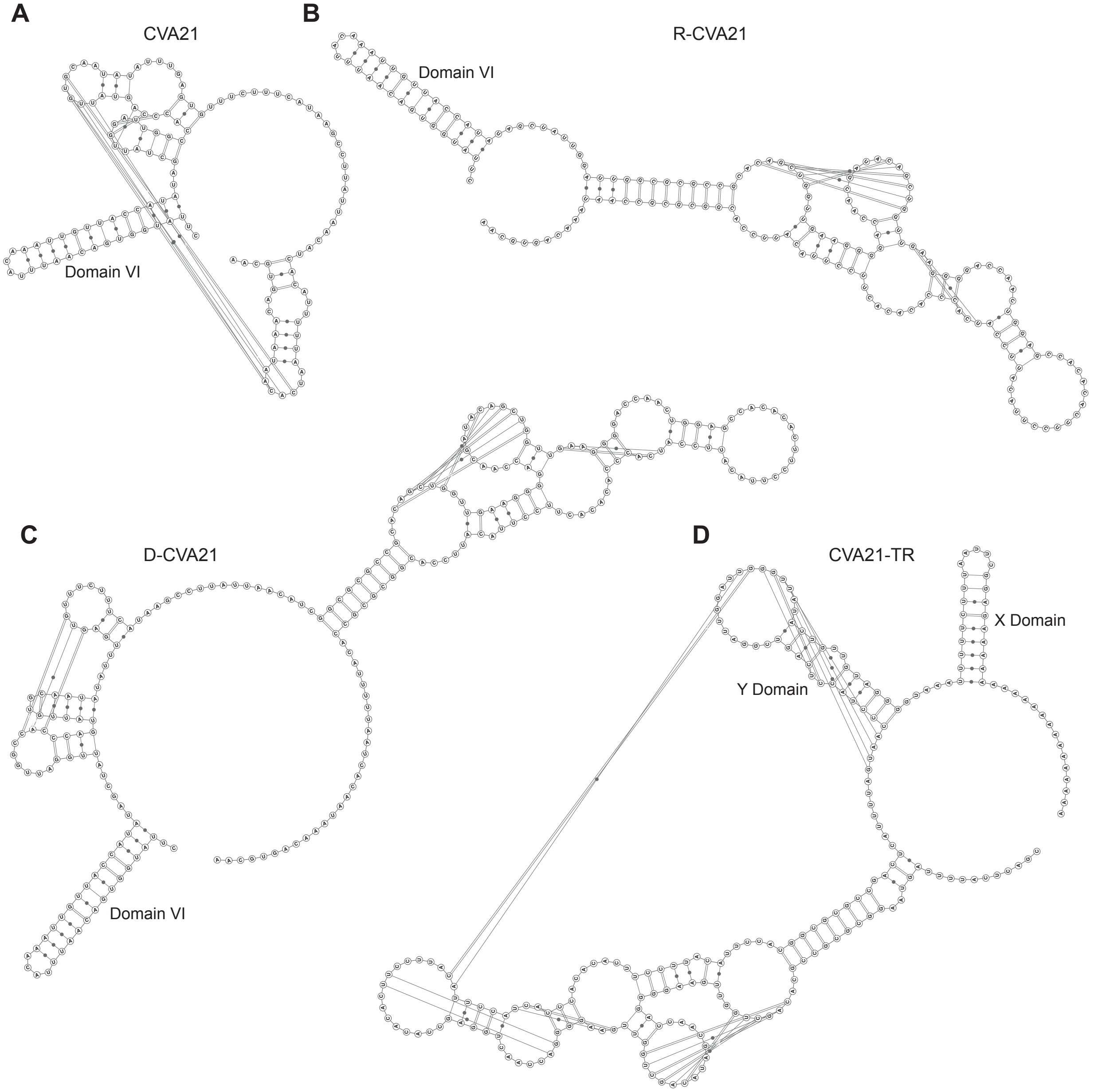

Figure S2. Predicted secondary RNA structures of microRNA-detargeted CVA21 NCRs. Predicted pseudoknot interactions are depicted by lines. Predictions of structures of the regions spanning domain VI and the downstream spacer in the 5’ NCR for CVA21 (**A**), R-CVA21 (**B**), D-CVA21 (**C**), or the 3’ NCR of CVA21-TR (**D**).

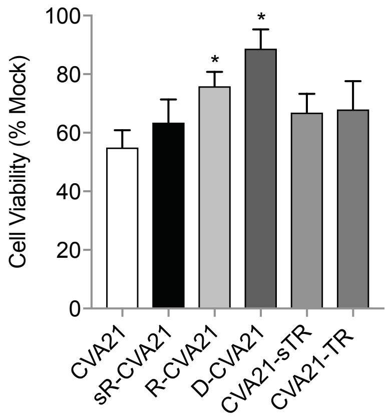

Figure S3. *In vitro* characterization of microRNA-detargeting efficacy in differentiated primary human skeletal muscle cells. Primary human skeletal muscle cells were incubated in differentiation media for 96 hrs. Differentiated cells were infected with unmodified or miRT-CVA21 at an MOI of 1. Cell viability was measured at 48 hrs post infection. This experiment was repeated in triplicate and the data are represented as mean cell viability +/- standard deviations. $^*p < 0.05$ compared to CVA21 infection was considered significant.

Virus	X	Y	Level	-Level	Score Mean Diff	Std Err Diff	Z	p-Value	Hodges-Lehmann	Lower CL	Upper CL	Difference Plot	Median	Significant
CVA21	Condition	% Alive	miR142	Mock Infection	1.6	1.914854	0.835573	0.8191	1.36209	-3.98812	8.547545	1.3620885	3.156059	N
CVA21	Condition	% Alive	miR-133	Mock Infection	0.4	1.914854	0.208893	0.9987	0.30378	-4.43435	5.277986	0.3037818	4.858669	N
CVA21	Condition	% Alive	miR-206	Mock Infection	-0.4	1.914854	-0.20889	0.9987	-0.11628	-4.74996	5.307162	-0.1162759	4.96664	N
CVA21	Condition	% Alive	Combination	Mock Infection	0.4	1.914854	0.208893	0.9987	1.20778	-4.12101	6.503142	1.2077765	3.338805	N
sR-CVA21	Condition	% Alive	miR142	Mock Infection	2	1.914854	1.044466	0.6792	1.66108	-7.13336	10.09741	1.6610836	3.455054	N
sR-CVA21	Condition	% Alive	miR-133	Mock Infection	-1.6	1.914854	-0.83557	0.8191	-0.93815	-18.0799	8.408737	-0.9381508	5.747349	N
sR-CVA21	Condition	% Alive	miR-206	Mock Infection	-4.8	1.914854	-2.50672	0.0421	-42.10847	-109.464	-22.8808	-42.108469	47.22461	Y
sR-CVA21	Condition	% Alive	Combination	Mock Infection	-4.8	1.914854	-2.50672	0.0421	-67.01642	-87.9534	-44.3064	-67.016417	72.13255	Y
R-CVA21	Condition	% Alive	miR142	Mock Infection	0	1.914854	0	1	0.34846	-3.83397	6.130802	0.3484557	4.102876	N
R-CVA21	Condition	% Alive	miR-133	Mock Infection	-4.8	1.914854	-2.50672	0.0421	-15.84674	-38.4094	-5.07339	-15.846737	18.7038	Y
R-CVA21	Condition	% Alive	miR-206	Mock Infection	-4.8	1.914854	-2.50672	0.0421	-92.19014	-101.639	-41.3396	-92.190139	95.0472	Y
R-CVA21	Condition	% Alive	Combination	Mock Infection	-4.8	1.914854	-2.50672	0.0421	-67.40677	-73.0015	-16.2039	-67.406772	70.47463	Y
D-CVA21	Condition	% Alive	miR142	Mock Infection	0	1.914854	0	1	0.29485	-5.22073	8.960566	0.2948469	4.302206	N
D-CVA21	Condition	% Alive	miR-133	Mock Infection	-3.6	1.914854	-1.88004	0.1838	-35.98738	-61.8388	3.144034	-35.987376	40.23144	N
D-CVA21	Condition	% Alive	miR-206	Mock Infection	-4.8	1.914854	-2.50672	0.0421	-106.95717	-120.608	-66.4003	-106.95717	110.4039	Y
D-CVA21	Condition	% Alive	Combination	Mock Infection	-4.8	1.914854	-2.50672	0.0421	-62.56788	-86.0686	-53.9022	-62.567882	63.65272	Y
CVA21-sTR	Condition	% Alive	miR142	Mock Infection	0.4	1.914854	0.208893	0.9987	0.44849	-3.43769	9.98751	0.4484926	3.372	N
CVA21-sTR	Condition	% Alive	miR-133	Mock Infection	-3.2	1.914854	-1.67115	0.2743	-5.56374	-15.4224	3.986601	-5.5637443	8.27973	N
CVA21-sTR	Condition	% Alive	miR-206	Mock Infection	-4.8	1.914854	-2.50672	0.0421	-44.88248	-55.5044	-11.4917	-44.882478	55.82902	Y
CVA21-sTR	Condition	% Alive	Combination	Mock Infection	-4	1.914854	-2.08893	0.1177	-49.29266	-60.8636	0.041527	-49.292655	54.64134	N
CVA21-TR	Condition	% Alive	miR142	Mock Infection	1.2	1.914854	0.62668	0.9246	1.12578	-3.65968	7.313814	1.1257801	3.629468	N
CVA21-TR	Condition	% Alive	miR-133	Mock Infection	-2.4	1.914854	-1.25336	0.5295	-3.45313	-10.1213	3.006561	-3.4531284	6.96632	N
CVA21-TR	Condition	% Alive	miR-206	Mock Infection	-2.4	1.914854	-1.25336	0.5295	-3.81484	-25.0441	3.347083	-3.8148401	4.983251	N
CVA21-TR	Condition	% Alive	Combination	Mock Infection	-2	1.914854	-1.04447	0.6792	-3.23346	-63.1956	3.928463	-3.2334609	5.423438	N

Table S1. Nonparametric Steel multiple comparisons with mock infection controls statistical analysis of microRNA targeting efficiency of samples described in Fig. 3A.
