# Supplementary material for: Oncolytic Activity of Targeted Picornaviruses Formulated as Synthetic Infectious RNA
Source: Mol Ther Oncolytics. 2020 May 19;17:484–95. doi: 10.1016/j.omto.2020.05.003 (PMC7276391; doi:10.1016/j.omto.2020.05.003)
Supplement: Document S2. Article plus Supplemental Information [file mmc2.pdf]

Molecular Therapy
Oncolytics
Original Article

Oncolytic Activity of Targeted Picornaviruses Formulated as Synthetic Infectious RNA

Noura B. Elsedawy,[1,2] Rebecca A. Nace,[1] Stephen J. Russell,[1] and Autumn J. Schulze[1]

[1]Department of Molecule Medicine, Mayo Clinic College of Medicine, Rochester, MN 55902, USA

Infectious nucleic acid has been proposed as a superior formulation for oncolytic virus therapy. Oncolytic picornaviruses can be formulated as infectious RNA (iRNA), and their unwanted tropisms eliminated by microRNA (miRNA) detargeting. However, genomic insertion of miRNA target sequences into coxsackievirus A21 (CVA21) iRNA compromised its specific infectivity, negating further development as a novel oncolytic virus formulation. To address this limitation, we substituted a muscle-specific miRNA response element for the spacer region downstream of the internal ribosomal entry site in the 5′ non-coding region of CVA21 iRNA, thereby preserving genome length while avoiding the disruption of known surrounding RNA structural elements. This new iRNA (R-CVA21) retained high specific infectivity, rapidly generating replicating miRNA-detargeted viruses following transfection in H1-HeLa cells. Further, in contrast with alternatively configured iRNAs that were tested in parallel, intratumoral administration of R-CVA21 generated a spreading oncolytic infection that was curative in treated animals without associated myotoxicity. Moreover, R-CVA21 also exhibited superior miRNA response element stability *in vivo*. This novel formulation is a promising agent for clinical translation.

INTRODUCTION

Although oncolytic viruses are proving safe, their efficacy as monotherapies is thus far inadequate. Among the most efficacious oncolytic viruses is a proprietary formulation of coxsackievirus A21 (CVA21; CAVATAK). The tolerability and efficacy of CVA21 has been demonstrated in phase I and II clinical trials.[1–5] However, these studies are limited to immune-competent patients $\geq$18 years of age, leaving the safety of CVA21, which has the potential to cause myositis in immunocompromised hosts, in pediatric/adolescent or severely immunocompromised patients largely unknown.[6,7] Additionally, clinical protocols can include up to 19 treatments, a significant financial burden that will continually increase with combination therapies. These studies set a precedent for developing a more cost-effective oncolytic CVA21 formulation with enhanced potency and safety to further improve clinical outcomes and expand the patient population eligibility.

Infectious nucleic acid (INA) has the potential to safely enhance the potency of CVA21 monotherapy while significantly reducing treatment costs. INA can be less immunogenic than virus particles, providing a mechanism to avoid neutralization during repeat dosing.[8] Entry of INA into a cell is not dependent upon a specific receptor increasing the potential for enhanced seeding of heterogeneous cell types within tumor beds, while maintaining the tumor specificity of the spreading oncolytic infection. Furthermore, manufacturing of INA may be simpler, more readily controlled to reduce heterogeneity among batches, and more cost-effective than producing clinical-grade virus.[9–12]

We previously demonstrated the feasibility of formulating CVA21 as INA by delivering infectious RNA (iRNA) encoding full-length genomes of CVA21 to mice bearing human myeloma xenografts.[13] The oncolytic activity of CVA21 iRNA delivered by intratumoral injection was equitable to CVA21 virus particles; however, both caused lethal myositis. The utility of microRNA (miRNA) detargeting to eliminate the unwanted toxicities of picornaviruses has been thoroughly established.[7,14–17] However, while picornaviruses can be rescued with high efficiency from RNA transcripts encoding their full-length genomes, perturbation of the viral genome can significantly alter this efficiency.[18] Hence, although insertion of a muscle-specific miRNA response element within the 3′ non-coding region (NCR) ameliorated the associated myotoxicity of CVA21 virotherapy,[7] it also eliminated all oncolytic activity of the iRNA.

The 3′ NCRs of enteroviruses contain a pseudoknot replication element known as the *ori*R that is involved in viral RNA synthesis and poly(A) tail elongation.[19–25] Because the entire 3′ NCR of CVA21 is predicted to be involved in the formation of the *ori*R, insertion of a miRNA response element within this region without disrupting this is highly improbable. Additionally, picornaviruses are known to have limited carrying capacities due to capsid rigidity, and insertion of heterologous sequences reduces particle stability.[26] This evolutionary constraint increases the probability of reversion mutant emergence, which can negate the miRNA-based safety element.[7] Although the majority of miRNA-detargeted oncolytic viruses have 3′ NCR configurations, we and others have shown that localization

Received 4 February 2020; accepted 12 May 2020;
https://doi.org/10.1016/j.omto.2020.05.003.

[2]Present address: Roswell Park Comprehensive Cancer Center, Buffalo, NY 14203, USA

Correspondence: Autumn J. Schulze, PhD, Department of Molecule Medicine, Mayo Clinic College of Medicine, 200 1st Street S.W., Rochester, MN 55902, USA.
E-mail: schulze.autumn@mayo.edu

This is an open access article under the CC BY license (http://creativecommons.org/licenses/by/4.0/).

within 5′ NCRs can also result in detargeting.[14,17] The 5′ NCRs of enteroviruses are predicted to contain six structural domains required for genome replication and translation.[27–34] Domains II–VI comprise the type I internal ribosome entry site (IRES) that regulates cap-independent translation. The IRES is followed by a long (~126–150 nt) variable linker designated the "spacer or ribosomal scanning region." Although its physiological significance to CVA21 is unknown, a previous study found that a 103-nt deletion in this region resulted in no attenuation of a mouse-pathogenic poliovirus mutant.[35] In this study, we show that replacement of the majority of the spacer region in CVA21 with a muscle-specific miRNA response element preserves virus replication, enhances miRNA target genetic stability *in vivo*, and maintains oncolytic activity of the miRNA-detargeted INA in the absence of toxicity.

RESULTS

Insertion of miRNA Response Element into the 3′ NCR Reduces Specific Infectivity and Eliminates Oncolytic Activity of CVA21 iRNA

CVA21-3′miRT, previously generated in our lab, contained a muscle-specific miRNA response element comprising two copies each of sequences completely complementary to miRNA 133 (miR-133) and miR-206, each separated by 4-6-nt-long linkers (133-133-206-206).[7] CVA21-3′miRT virus replicated similarly to unmodified CVA21 and maintained oncolytic activity in the absence of toxicity. However, infectious virus recovery following transfection of *in vitro*-derived iRNA encoding CVA21-3′miRT was severely delayed in both H1-HeLa and Mel624 cells (Figure 1A). Consequently, CVA21-3′miRT iRNA did not induce viremia, exhibit any oncolytic activity, or enhance survival of severe combined immunodeficiency (SCID) mice bearing subcutaneous Mel624 xenografts (Figures 1B and 1C). The miRNA response element of CVA21-3′miRT was inserted downstream of the polyprotein stop codon at the base of the first predicted stem loop involved in *ori*R formation. RNA secondary structural prediction (including pseudoknots) demonstrate the disruption of this stem loop and reduced probability of the pseudoknot formation (Figure 1D).

Specific Infectivity of CVA21-3′miRT Is Temperature Sensitive and Involves Poly(A) Tail Elongation Function of *ori*R

No single mutation or combination of mutations could be identified to compensate for the *ori*R impediment in the recovered virus stocks of CVA21-3′miRT. Therefore, we hypothesized that the reduced specific infectivity of CVA21-3′miRT iRNA was based on the instability of the *ori*R and a consequential delay in poly(A) tail elongation. In support of our hypotheses, we found that the delay in CVA21-3′miRT recovery from iRNA was significantly improved at 32°C (Figure S1A). In addition, working titers of CVA21-3′miRT could be generated at 37°C more rapidly if the *in vitro*-derived iRNAs were polyadenylated prior to transfection (Figure S1B). These data support the existence and importance of an *ori*R structure in CVA21 replication and emphasize the need for optimized configuration of miRNA response elements in iRNA formulations of oncolytic viruses.

Replacement of the Spacer Region with the miRNA Response Element Minimizes Potential Structural Alterations

In contrast with the complex structural environment of the 3′ NCR, the 5′ NCR of CVA21 is predicted to contain a disordered spacer region directly downstream of the IRES. The role of this domain in CVA21 replication is unknown. Although deletions in this region have been shown to be tolerated in a mouse-pathogenic poliovirus *in vitro* and *in vivo*, it has been indicated in binding an IRES *trans*-acting factor that enhances enterovirus 71 translation.[35,36] We generated a construct wherein residues 631–698 in the spacer region of the CVA21 5′ NCR were exchanged with our muscle-specific miRNA response element (R-CVA21). RNA secondary structural analysis predicted that this configuration resulted in maintenance of domain VI within the IRES (Figures S2A and S2B).

For comparative analyses, we generated a panel of various 5′ and 3′ miRNA-detargeted CVA21 genome configurations. We hypothesized that elongation of the genome would decrease the genetic stability of the miRNA response element; therefore, we constructed a CVA21 genome with our muscle-specific miRNA response element directly inserted into the spacer region at nucleotide position 686 (D-CVA21). This region was chosen based on RNA structural modeling that demonstrated response element insertion at this position also minimized disruption of domain VI in the IRES (Figure S2C). For additional analysis of the pressure exerted by genome elongation and to compare 5′ to 3′ configurations, we generated a new 3′ miRNA-detargeted CVA21. Direct insertion of the miRNA response element anywhere within the 3′ NCR inevitably resulted in destabilized *ori*R formation predictions. In order to generate a 3′ NCR miRNA-detargeted configuration that maintained *ori*R formation, we duplicated residues 7325–7340 comprising the junction between the end of the viral open reading frame and the 3′ NCR, and inserted the miRNA response element between these "terminal repeats" (CVA21-TR). As shown in Figure S2D, inclusion of this "stabilizing domain" restores formation of the predicted *ori*R stem loops, but may not be optimal for pseudoknot formation. Finally, we generated two constructs to analyze the required length of the miRNA response element at these new locations. These two constructs contained a miRNA response element encoding only a single copy each of the sequences recognized by miR-133 and miR-206. This short miRNA response element was encoded either within the 3′ NCR containing the new stabilizing domain (CVA21-sTR) or replaced the spacer region within the 5′ NCR (sR-CVA21). Figure 2A depicts the CVA21 viral genome, the miRNA response elements used, and all sites of insertion that were tested.

R-CVA21 iRNA Maintains Specific Infectivity Similar to Unmodified CVA21 iRNA

In vitro-derived iRNA transcripts encoding each of the miRNA-detargeted CVA21 genomes were transfected into H1-HeLa or Mel624 cells, and the rate of virus recovery was evaluated (Figure 2B). The specific infectivity of all three 5′ NCR-targeted CVA21 genomes was similar to unmodified CVA21 iRNA. A slight lag in virus recovery rate was noted for R-CVA21 in H1-HeLa cells, but not in Mel624

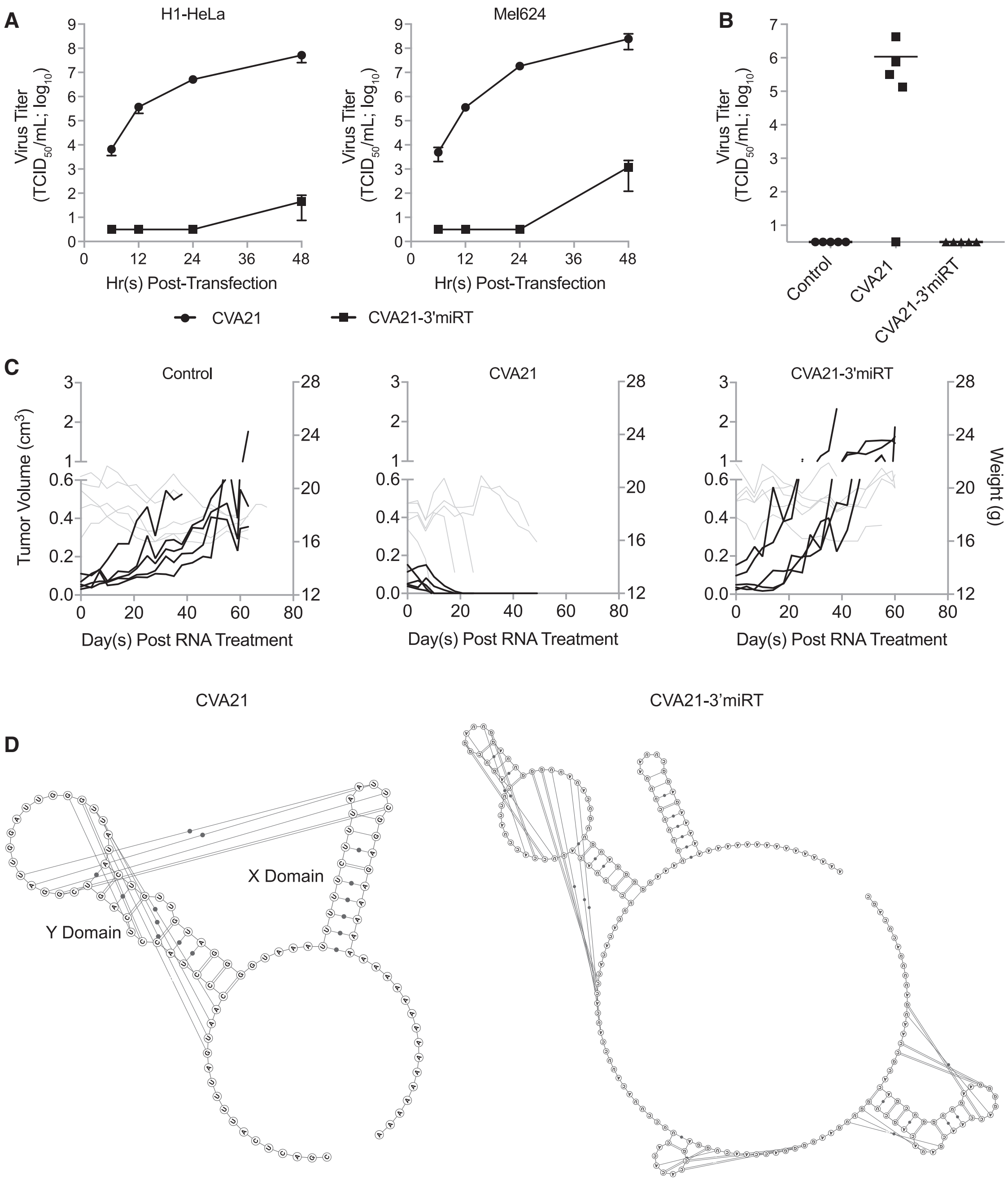

Figure 1. CVA21-3′miRT Insertion Site Disrupts *ori*R Structure Resulting in Reduced Specific Infectivity and Oncolytic Activity of iRNA

(A) Infectious virus recovery time course in H1-HeLa and Mel624 cells. Cells were transfected with 1 μg *in-vitro*-derived RNA transcripts encoding CVA21 or CVA21-3′miRT genomes. Samples were collected at various times posttransfection and infectious virus titrated. The experiment was run in triplicate, and data are represented as mean viral titers ± standard deviations. (B and C) CB17 ICR-SCID mice bearing subcutaneous Mel624 xenografts were treated intratumorally with saline (n = 5) or 30 μg iRNA encoding CVA21 (n = 5) or CVA21-3′miRT (n = 5). (B) Viral titers in sera collected from mice on day 7 posttreatment. (C) Tumor volumes (black lines) and weights (gray lines) of all treated mice. (D) Predicted secondary structures of CVA21 and CVA21-3′miRT 3′ NCRs. Stem loops Y and X are labeled, and the predicted pseudoknot interactions are depicted by lines.

cells. Although significantly improved compared with CVA21-3′miRT, both CVA21-TR and -sTR iRNAs exhibited delays in virus recovery following transfection in H1-HeLa cells. This deficiency was more prominent for CVA21-TR, suggesting that the terminal repeat cannot completely restore *ori*R stability in the presence of longer response elements.

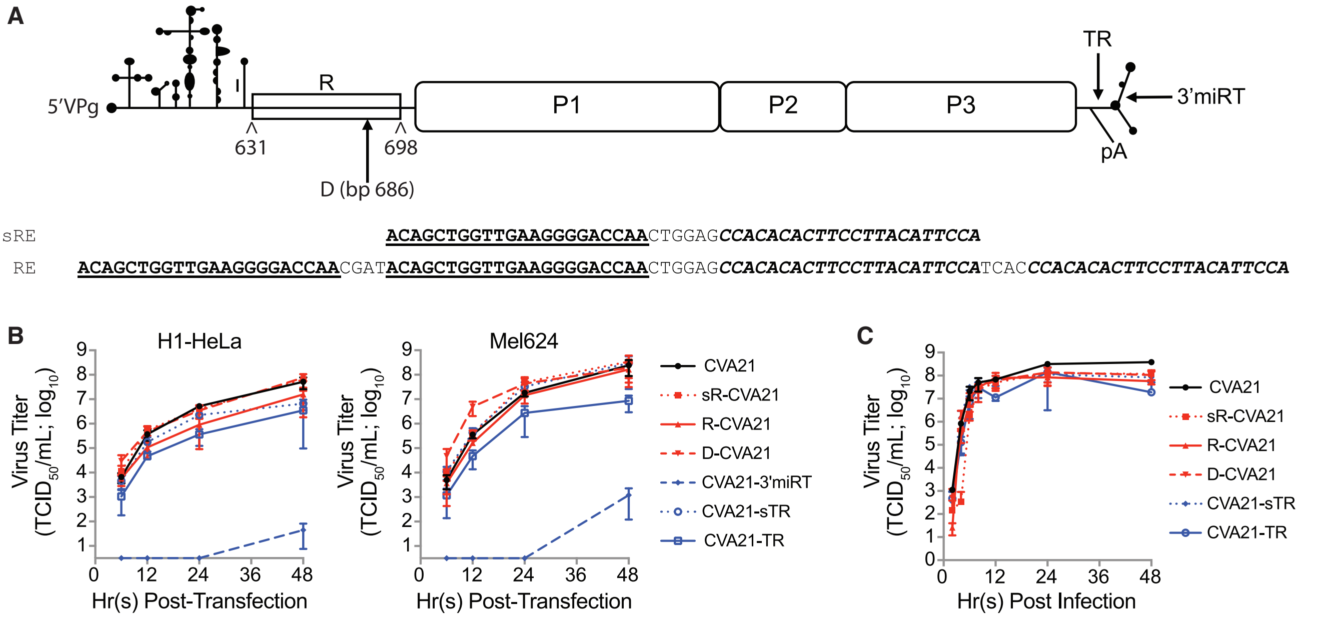

Figure 2. *In Vitro* Characterization of MicroRNA-Detargeted CVA21

(A) Schematic representation of the CVA21 genome and location and sequence of the muscle-specific microRNA response elements (REs) analyzed. (B) Infectious virus recovery time course of all iRNAs in H1-HeLa and Mel624 cells. Cells were transfected with 1 μg *in-vitro*-derived RNA encoding microRNA-detargeted genomes. Samples were collected at various times posttransfection and infectious virus titrated. The experiment was run in triplicate, and data are represented as mean viral titers ± standard deviations. (C) One-step growth curve analysis of microRNA-detargeted CVA21 viruses versus wild-type in H1-HeLa cells. Cells were infected at an MOI of 3. Samples were collected at various times postinfection and total virus titrated. The experiment was run in triplicate, and data are represented as mean viral titers ± standard deviations.

R-CVA21 Virus Replication Is Unaltered in Tumor Cells

Single-step growth curve analysis was conducted to characterize the replication capacity of the miRNA-detargeted viruses. H1-HeLa cells, which do not express miR-133 or miR-206, were infected at an MOI of 3. Samples were harvested at various times postinfection, and total accumulation of virus was titrated. As shown in Figure 2C, all of the newly constructed miRNA-detargeted CVA21 viruses replicated similar to unmodified CVA21, although a slight delay was observed for sR-CVA21. These data are consistent with the previous observation that CVA21-3′miRT virus replicated similar to wild-type CVA21 even though a significant delay in virus rescue from *in vitro*-derived transcripts was observed.

R-CVA21 miRNA Targeting Efficacy Is Enhanced Compared with 3′ Configurations

To evaluate the efficiency and specificity of miRNA detargeting, we measured cell viability and virus replication in H1-HeLa cells transfected with complementary or control synthetic miRNA mimics (Figures 3A and 3B). At 12 h post-transfection, the cells were infected with unmodified or miRNA-detargeted CVA21 at an MOI of 1. At 24 h postinfection, the virus titer in the supernatant was determined and the cells were assayed for proliferation as a measure of viability. Viruses with 5′ NCR localized response elements were more readily controlled than 3′ NCR insertions. Virus replication was minimally controlled by miR-133 for R-CVA21 and D-CVA21. miR-206 was much more efficient at controlling virus replication, resulting in increased cell viability and decreased virus titers for all three 5′-detargeted viruses, and to a lesser extent for CVA21-sTR. Virus tropism was similarly regulated in H1-HeLa cells transfected with both miR-133 and miR-206 in combination. CVA21-TR replication was not regulated by miR-133 or miR-206, and regulation with a combination of both mimics was inefficient and highly variable. No difference in cell viability and virus titer was observed in H1-HeLa cells transfected with the miRNA-142 control mimic or non-transfected cells. Unfortunately, there is no convenient cell culture system for differentiated muscle cells that allow the evaluation of miRNA targeting *in vitro*. This is in part due to a weak induction of muscle-specific RNAs in cultured muscle stem cells after *in vitro* differentiation and poor susceptibility of cultured muscle cells to CVA21 killing. Nevertheless, to confirm that our viruses are indeed impacted by naturally occurring muscle-specific miRNAs, we infected differentiated primary human skeletal muscle cells at an MOI of 1 and determined the cytotoxicity of the unmodified and miRT-CVA21 at 48 h postinfection. All miRT-CVA21 variants had reduced cytopathic effects (CPEs) in differentiated primary human skeletal muscle cells *in vitro*, but only R-CVA21 and D-CVA21 reached significance with $p = 0.0133$ and $p = 0.0003$, respectively (Figure S3).

R-CVA21 Ameliorates Toxicity in Tumor-Bearing Mice

To evaluate the therapeutic index of R-CVA21, we treated female CB17 ICR-SCID mice bearing subcutaneous Mel624 xenografts by

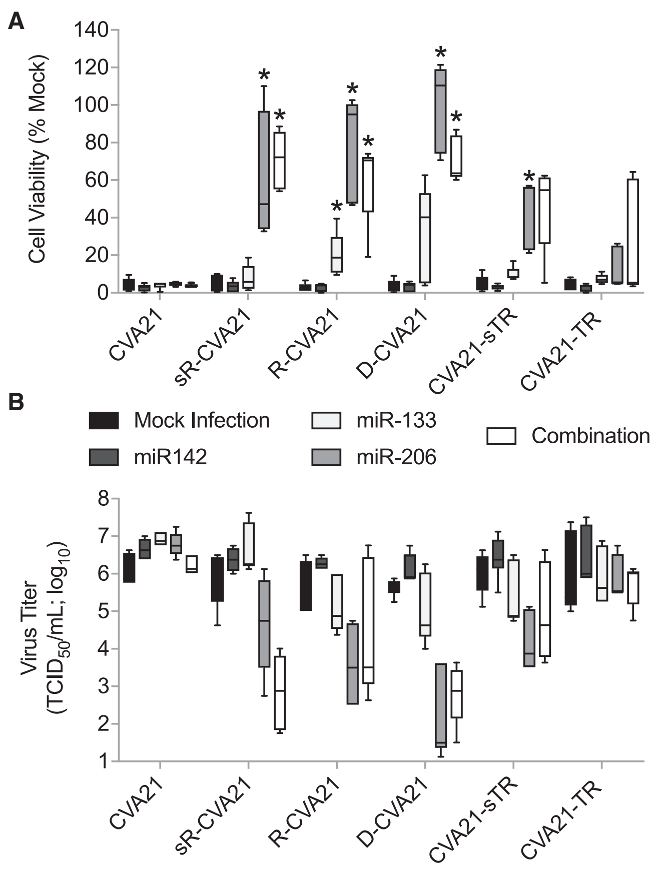

Figure 3. *In Vitro* Characterization of MicroRNA-Detargeting Specificity and Efficacy

H1-HeLa cells were transfected with 100 nM microRNA mimics and infected 12 h later with unmodified or miRT-CVA21 at an MOI of 1. (A and B) Cell viability (A) and virus titer in the supernatant (B) were measured 24 h postinfection. This experiment was repeated in duplicate (n = 2; with five distinct technical replicates), and the data are represented as interleaved minimum and maximum box and whisker plots with median marks. *$p < 0.05$ compared with mock infection controls.

intratumoral injection of 30 μg *in vitro*-derived iRNA encoding each of the miRNA-detargeted CVA21 genomes. Based on our *in vitro* data, we did not continue evaluation of CVA21-TR. As shown in Figure 4A, control saline-treated animals all exhibited progressive tumor growth and were euthanized due to tumor volume exceeding 10% body weight or tumor ulceration. Rapid tumor regression was observed in all mice treated with iRNA encoding unmodified or miRNA-detargeted CVA21 (Figure 4A). However, toxicity in the form of hindlimb paralysis (HLP), sudden death (FD), or excessive weight loss (WL) was observed in all mice treated with unmodified CVA21 iRNA and a proportion of mice treated with all miRNA-detargeted CVA21 iRNAs, except R-CVA21. All R-CVA21-treated mice appeared healthy and tumor-free at the end of the study, resulting in a significant increase ($p < 0.05$) in overall survival compared with all other groups (Figure 4B). In contrast with that observed in mice treated with CVA21-3′\miRT, infectious virus could be isolated from the sera of mice treated with R-CVA21 at 7 days postinjection at levels similar to that found in mice treated with unmodified CVA21 iRNA (Figure 4C).

A Threshold Level of Delivery Is Required for Therapeutic Efficacy of R-CVA21 iRNA

After validating the ability of R-CVA21 to initiate a successful and safe oncolytic infection, we sought to determine the minimum dose of iRNA required to mediate tumor regression. We conducted a dose-response study in female CB17 ICR-SCID mice bearing subcutaneous Mel624 xenografts. Tumor-bearing animals were treated with doses of 1, 4, 8, 16, or 32 μg *in vitro*-derived R-CVA21 iRNA via intratumoral injection. As shown in Figure 5A, control-treated mice exhibited progressive tumor growth, and all were euthanized because of tumor size or ulceration. Four of five mice treated with 1 μg R-CVA21 iRNA also had progressive tumor growth; however, complete tumor regression was observed in one mouse. All mice treated with RNA doses of 4, 8, 16, or 32 μg exhibited rapid tumor regression similar to results previously reported following intratumoral injection of the same doses of RNA encoding wild-type CVA21.[13] One mouse treated with 8 μg R-CVA21 RNA developed dual HLP 28 days posttreatment. No infectious virus was recovered from the skeletal muscle of this mouse. Viral genomes could be recovered from the skeletal muscle of the paralyzed limb with nested PCR, but sequence analysis did not show any mutations in the response element. This mouse had lower viremia than three of her four group mates at day 9 posttreatment and had no palpable subcutaneous tumor by day 10 posttreatment. Toxicity in this animal could possibly be due to compression of the spinal cord by undetectable tumor; however, based on our previous studies, it is most likely due to myositis associated with viral infection.[7,13] Another mouse treated with 32 μg R-CVA21 iRNA was found dead on day 78 after iRNA treatment despite routine monitoring. All but three mice treated with ≥4 μg R-CVA21 iRNA displayed high viral loads in sera on day 9 posttreatment (Figure 5B). Of the mice treated with 1 μg R-CVA21 iRNA, high viral loads were observed only in the mouse that displayed complete tumor regression (Figure 5B). All treatment groups except the 1-μg-treated mice had improved overall survival compared with control-treated mice. Groups treated with 4, 16, and 32 μg R-CVA21 iRNA had significantly improved overall survival compared with control-treated mice with $p = 0.0029$, 0.0029, and 0.0081, respectively (Figure 5C). Although animals treated with 8 μg R-CVA21 iRNA had improved survival, this group failed to reach statistical significance ($p = 0.0528$).

Replacement of Spacer Region Enhances miRNA Response Element Stability

At the time of euthanasia, viral genomes were isolated from the sera and skeletal muscle of all R-CVA21-treated mice and mice with clinical signs of toxicity, and sequence analysis was performed. Reversion mutants were detected in the skeletal muscle of one mouse treated with sR-CVA21 that developed HLP and in all three mice treated with D-CVA21 that developed HLP. In contrast, no reversion or escape mutants were detected in any of the mice treated with R-CVA21. Of note, reversion mutants were also detected in both of

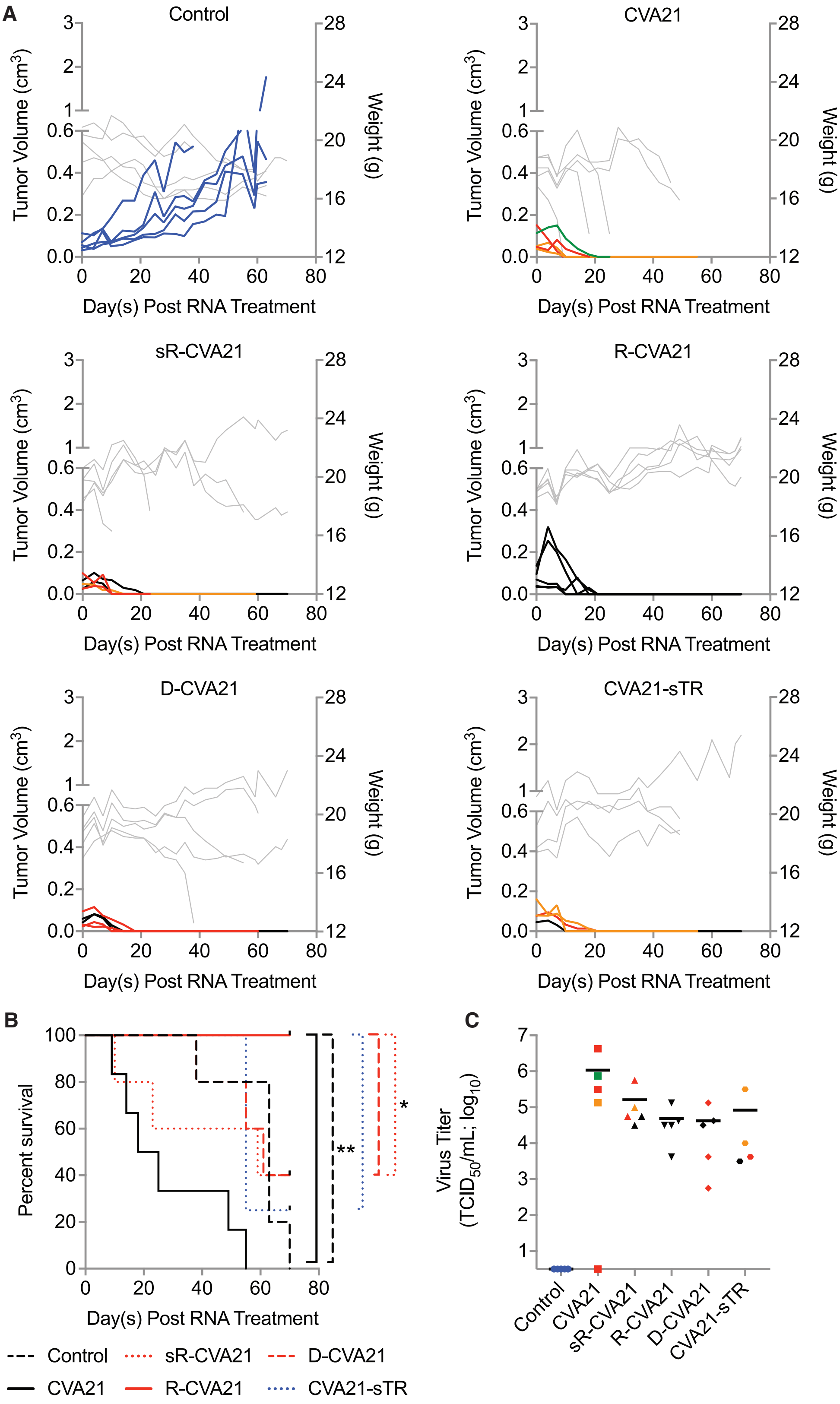

(legend on next page)

the evaluable mice treated with CVA21-sTR. In an effort to determine the stability of the miRNA response elements *in vitro*, we performed serial passage of each miRNA-detargeted CVA21 in differentiated TE671 (dTE671) muscle cells that express miR-133 and miR-206. dTE671 cells were initially infected at an MOI of 10. At 24 h postinfection, the samples were collected and fresh dTE671 cells infected with 50% of the clarified lysates. Viral RNA was isolated from cleared lysates of seven serial passages, cDNA synthesized, and regions containing the response elements amplified for sequencing. In both experiments, reversion mutants were detected in D-CVA21 samples two to three passages prior to detection in R-CVA21 and sR-CVA21 samples. These data, in conjunction with the *in vivo* isolation rate of reversion mutants from D-CVA21- versus R-CVA21-treated mice, suggest that elongation of the spacer region with direct miRNA response element insertion decreases the genetic stability.

DISCUSSION

CVA21 monotherapy has been shown to be well tolerated in patients and to target various tumor types, including melanoma, non-small cell lung cancer, and bladder cancer.[3,5,37–40] However, the overall efficacy has been moderate, and efforts to augment clinical outcomes are centered on combination with immunotherapeutics. Preclinical attempts to enhance the potency of CVA21 monotherapy are focused on genetic engineering of the viral genome and methods to enhance delivery. In all cases, the potential for off-target toxicities increases, as does the cost of therapy. Currently, clinical protocols are composed of repeat injections of CVA21 up to 19 total treatments. The cost of generating this quantity of virus per patient is significant, and efficacy is dependent upon tumor cells expressing the virus receptor human intercellular adhesion molecule I (hICAM-1). Additionally, patients develop antiviral antibodies during treatment, and the impact of these antibodies on therapeutic efficacy has not been fully elucidated. Using INA in lieu of virus particles could significantly reduce the cost of manufacturing the clinical product and may allow the introduction of viral RNA into tumor cells that do not express hICAM-1, expanding the initial distribution of the virus, while maintaining the hICAM-1-mediated tumor specificity of the progeny virions.[7,13,41–45] Furthermore, INA can be less immunogenic than virus particles, increasing the potential for initiation of an oncolytic phase during repeat administrations, which could increase the potency of CVA21 monotherapy.[8] These and other potential benefits of iRNA formulations for oncolytic viruses remain to be determined and may depend in part upon the development of non-viral delivery vehicles capable of efficient systemic delivery of iRNA to the tumor bed.

No matter the mechanism of increasing the potency of CVA21 therapy, the necessity of ensuring safety is constant. Although CVA21 has been shown to be well tolerated, we emphasize that the current clinical studies are conducted in patients over the age of 18 years with functioning immune systems. Nearly all immunodeficient tumor-bearing mice succumb to CVA21-induced myotoxicity, making relevant preclinical evaluation of any CVA21 recombinant impossible. Furthermore, although CVA21 has demonstrated activity against a variety of cancers, including multiple myeloma, clinical studies in these patients, as well as severely immune compromised and pediatric patients, are lacking. Development of a miRNA-detargeted CVA21 that maintains the biological properties and oncolytic activity of an unmodified CVA21 makes this a possibility.

Although the therapeutic efficacy of CVA21 iRNA has been shown to be equitable to CVA21 virus particles in xenograft mouse tumor models, obtaining the same for a miRNA-detargeted CVA21 INA has remained elusive.[13] Picornaviruses are particularly amenable to miRNA detargeting; however, because their genomes are short and packed with RNA structural elements, localization of response elements that maximize target accessibility and stability without disrupting viral replication is difficult. We show that the 5′ NCR is more amenable to miRNA target insertion, and exchanging the spacer region with the response element increases genetic stability of the insert *in vivo*. Compared with the 3′ NCR miRNA-detargeted constructs, the 5′ NCR-detargeted viral genomes had enhanced specific infectivity and targeting efficacy. Although separation of the miRNA response element from the *ori*R by repeating nucleotides predicted to be involved in *ori*R stabilization enhanced the specific infectivity of the iRNA, it did not improve stability *in vivo* and decreased the accessibility of the longer response element. We show, for the first time, that a miRNA-detargeted iRNA (R-CVA21) is able to mount a successful oncolytic infection in a preclinical melanoma xenograft mouse model similar to wild-type CVA21 iRNA without causing toxicity. Our studies emphasize the importance of ensuring the miRNA response element insertion does not alter the properties of the viral genome, and that the response element is stable to obtain successful results. Future studies are needed to determine the immunogenic and oncolytic potential of R-CVA21 iRNA compared with the virus formulation in immune-competent animals, including the value of combination with immunotherapies both from a therapeutic and a mechanistic standpoint. Subsequent studies are also needed to determine the ability of termini modifications to enhance stability and translatability of the iRNA and their overall effects on therapeutic efficacy.

The targeting strategy described here has the potential to be applied to other picornaviruses with similar IRESs, provided the spacer region is not involved in binding IRES-transacting factors and the response

Figure 4. R-CVA21 iRNA Mounts a Spreading Oncolytic Infection in Mice Bearing Mel624 Xenografts without Causing Toxicity

CB17 ICR-SCID mice bearing subcutaneous Mel624 xenografts were treated intratumorally with saline (n = 5) or 30 μg RNA encoding unmodified CVA21 (n = 5), sR-CVA21 (n = 5), R-CVA21 (n = 5), D-CVA21 (n = 5), or CVA21-sTR (n = 4). (A) Tumor volumes (black or colored lines) and weights (gray lines) of all treated mice. (B) Kaplan-Meier survival graphs of the mice in (A). Overall survival comparisons were based on log rank statistics with *p $\leq$ 0.05 and **p $\leq$ 0.01 considered significant. Clinical toxicities observed are represented by colored lines or symbols in (A) and (B) as follows: hindlimb paralysis (red), sudden death (orange), weight loss (green), and tumor ulceration (blue). (C) Viral titers in sera collected from mice in (A) on day 7 posttreatment. Horizontal lines represent mean viral titers.

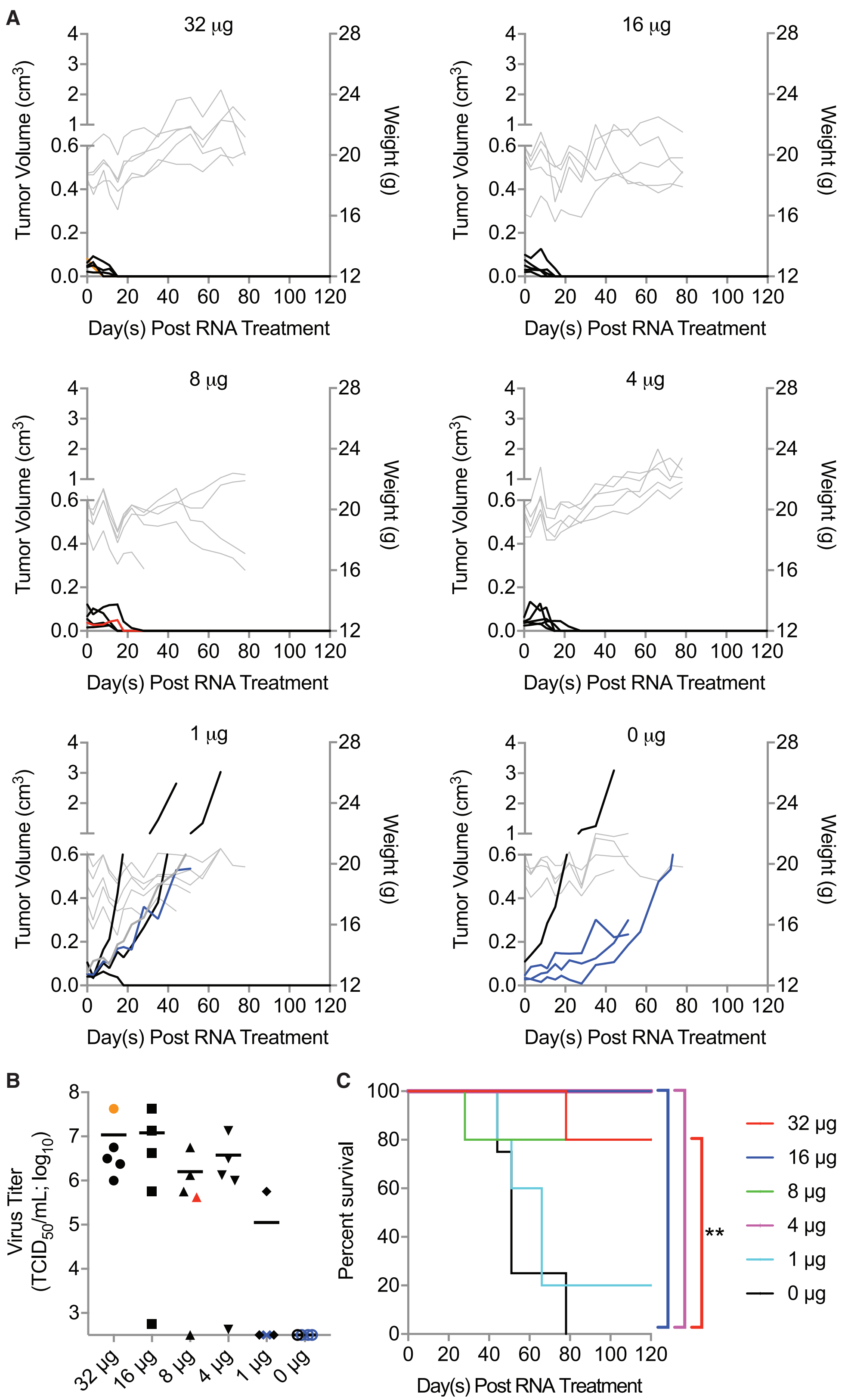

(legend on next page)

element does not encode a cryptic AUG site. Oncolytic picornaviruses are emerging as highly efficacious anticancer therapies including a CVA21, a chimeric polio-rhinovirus (PVSRIPO), and a non-pathogenic melanoma-adapted enteric cytopathic human orphan type 7 virus (Rigvir). miRNA-detargeted INAs encoding these clinically validated picornaviruses could significantly enhance the potential of these therapeutics to be used in patients with compromised immune systems, at increased doses, and at significantly reduced cost. Additionally, determining a universal mechanism for efficient miRNA detargeting of picornaviruses would greatly facilitate investigation of virus biology and developing combative treatments. We have developed the first miRNA-detargeted iRNA with activity equitable to a clinically proven oncolytic virus. This formulation has the potential to be revolutionary, providing a cheaper, easier, and safer platform for delivering oncolytic CVA21 to an expanded cohort of patients warranting clinical translation.

MATERIALS AND METHODS

Study Design

The objective of this research was to develop an INA encoding a miRNA-detargeted CVA21 genome that maintains oncolytic activity in the absence of toxicity. We hypothesized that incorporation of the miRNA response element into the 5′ NCR and maintenance of the overall genome length would minimize disruption of RNA structural elements and maximize miRNA response element stability, resulting in a superior formulation. A panel of CVA21 genomes with variable miRNA response element configurations was engineered and characterized to test our hypotheses. Experiments evaluating the specific infectivity of *in vitro*-derived RNA transcripts and those analyzing viral replication kinetics were conducted with three biological replicates based on previous analyses conducted in the same cell lines. The specificity and efficacy of each targeting unit in the miRNA response element was analyzed using cell viability assays and virus titration conducted with multiple technical and biological replicates for reproducibility. To evaluate the *in vivo* antitumor activity of our *in vitro*-derived RNA transcripts, we used mice (CB17 ICR-SCID mice), and the sample size (n = 5) was selected on the basis of previous analyses conducted in the same model and of sufficient power to determine statistically significant differences in tumor response rates and overall survival between treatment groups. Data collection was ceased and the mice euthanized if they exhibited $\geq$20% WL of their initial body weight, if their tumor volume reached $\geq$10% of their total body weight, if they ulcerated, or if they displayed clinical signs of myositis including HLP. Mice with palpable tumors were randomly assigned to treatment groups, and the investigators were not blinded. Sample collection, treatment, and processing information are included in the Results section, figure legends, or in other sections of the Materials and Methods. No outliers were excluded in the studies.

Cell Culture

H1-HeLa American Type Culture Collection (ATCC) CRL-1958, TE671 ATCC HTB139-1062, and primary human skeletal muscle ATCC PCS-950-010 cell lines were purchased from ATCC (Manassas, VA, USA). The human melanoma cell line Mel624 CL-IM118 was purchased from Imanis Life Sciences (Rochester, MN, USA). H1-HeLa and TE671 cells were grown in DMEM supplemented with 10% fetal bovine serum (FBS). Mel624 cells were grown in RPMI 1640 supplemented with 10% FBS. Both media were additionally supplemented with 100 U/mL penicillin and 100 μg/mL streptomycin, and both cell lines were grown at 37°C in 5% CO_2. Primary human skeletal muscle cells were grown in Mesenchymal Stem Cell Basal Medium (ATCC PCS-500-030) supplemented with Primary Skeletal Cell Muscle Growth Kit (ATCC PCS-950-040). Primary human skeletal muscle cells were differentiated in Primary Skeletal Differentiation Tool medium (ATCC PCS-950-050) according to the manufacturer's instructions for 96 h. Primary human skeletal muscle cells were grown at 37°C in 5% CO_2. The H1-HeLa cells were authenticated by ATCC using short tandem repeat DNA profiling. ATCC routinely tests morphology, karyotype, and species; however, we performed no further authentication of this cell line. The Mel-624 cells were authenticated by Imanis and certified free of interspecies cross-contamination by short tandem repeat (STR) profiling with nine STR loci. All cell lines were tested 48 h after thawing for mycoplasma contamination using a Universal Mycoplasma Detection Kit (ATCC; 30-1012K), and all cell lines routinely tested negative. All experiments were carried out within 3 months of cell thawing.

Plasmid Construction

AscI restriction enzyme sites were inserted into pGEM-CVA21 at the locations described by overlap-extension PCR, by site-directed mutagenesis, or by synthesizing fragments of the genome followed by subcloning into the full-length construct. miRNA sequences were obtained from Sanger Institute miRBase. Oligonucleotide ultramers encoding the response elements flanked by the overhang sequences generated during an AscI enzymatic digestion were annealed in T4 DNA ligase buffer (B0202S; NEB; Ipswich, MA, USA) by heating to 85°C and slowly cooling to 25°C. Annealed ultramers were ligated into the appropriately digested and purified AscI full-length vectors. Insertion site residue numbering is based on the unmodified pGEM-CVA21 plasmid. For R constructs, residues 631–698 within the spacer

Figure 5. Threshold Level of R-CVA21 iRNA Delivery Is Required for Tumor Destruction

CB17 ICR-SCID mice bearing subcutaneous Mel624 xenografts were treated intratumorally with saline (n = 4) or 32 μg (n = 5), 16 μg (n = 5), 8 μg (n = 5), 4 μg (n = 5), or 1 μg (n = 5) R-CVA21 iRNA. (A) Tumor volumes (black or colored lines) and weights (gray lines; not taken at end of study) of all treated mice. (B) Viral titers in sera collected from mice in (A) on day 9 posttreatment. (C) Kaplan-Meier survival curves of the mice in (A). Overall survival comparisons were based on log rank statistics with *p $\leq$ 0.05 and **p $\leq$ 0.01 considered significant. Clinical toxicities observed are represented by colored lines or symbols in (A) and (B) as follows: hindlimb paralysis (red), sudden death (orange), tumor ulceration (blue), and moribund (gray).

region of the 5′ NCR were deleted and replaced with an AscI site. The integrity of the targets was verified by sequencing.

iRNA Preparation

Plasmids were linearized with restriction endonuclease *MluI-HF* (NEB; R3198S), followed by ethanol precipitation and resuspension in nuclease-free water. *In vitro*-derived RNA transcripts were produced with a MEGAscript T7 transcription kit (AM1334; Thermo Fisher Scientific, Waltham, MA, USA) and purified with a MEGAclear transcription clean-up kit (AM1908; Thermo Fisher Scientific). For artificial poly(A) elongation, purified RNA transcripts were polyadenylated with a poly(A) tailing kit (AM1350; Thermo Fisher Scientific) according to the manufacturer's instructions. Purity and integrity of the transcripts were verified by gel electrophoresis (FlashGel RNA System, 57067; Lonza, Basel, Switzerland).

Virus Rescue and Sequencing

A total of 2×10^5 H1-HeLa cells were plated in a 12-well plate 24 h prior to transfection. To produce live virus, 1 μg *in vitro*-derived RNA was transfected per well using Mirus TransIT-mRNA transfection kit (miR 2250; Mirus Biosciences; Madison, WI, USA) according to the manufacturer's instructions. Once CPEs were apparent (48–72 h), the cells were scraped into the supernatant and the samples collected. Samples were subjected to three freeze-thaw cycles, the cellular debris removed by centrifugation, and the cleared lysate filtered through a 0.22-μm filter and passaged onto fresh H1-HeLa cells. Viral RNA was isolated from all virus stocks using a QIAamp viral RNA mini kit (52904; QIAGEN; Valencia, CA, USA) according to the manufacturer's instructions. Regions containing the response elements were amplified using the Titan one-tube reverse transcription-PCR system (11855476001; Roche Applied Science, Indianapolis, IN, USA), and the integrity of the inserts was verified by Sanger sequencing.

Virus Titration

A total of 1×10^4 H1-HeLa cells were seeded per well into 96-well plates and grown at 37°C in 5% CO_2. At 24 h, 10-fold serial dilutions of each virus stock were made, and 100 μL of each dilution added to each of eight replicate wells. The cells were incubated at 37°C in 5% CO_2 for 72 h. The cells were visually assessed for CPEs and scored as positive or negative. The 50% tissue culture infectious dose ($TCID_{50}$) per mL was calculated using the Spearman and Kärber equation.

Virus and RNA Growth Curves

A total of 2.5×10^5 H1-HeLa or Mel624 cells were seeded per well into 12-well plates and grown at 32°C or 37°C in 5% CO_2. At 24 h, each well was transfected with 1 μg *in vitro*-derived RNA transcripts or infected at an MOI of 3. Distinct samples were collected at specific times postinfection (2, 4, 6, 8, 12, 24, and 48 h) or transfection (6, 12, 24, and 48 h) and stored at −80°C. Following the completion of all time points, samples were frozen and thawed three times, and cellular debris was cleared from the lysates by centrifugation. The cleared lysates were then titrated as described above.

miRNA Targeting Assays

miRIDIAN miRNA mimics and a negative-control mimic corresponding to a *Caenorhabditis elegans* miRNA were purchased from Dharmacon (Lafayette, CO, USA). miRNA mimics were reverse transfected into H1-HeLa cells using the Mirus TransIT-mRNA transfection kit (miR 2250; Mirus Biosciences) at a concentration of 100 nM per well. In brief, transfection complexes were assembled in a 96-well plate and incubated at room temperature for 5 min. H1-HeLa cells in T75 flasks were trypsinized, counted, and resuspended in complete growth media at a concentration of 1×10^4 cells per 90 μL. 90 μL of cells was added per well, and the cells/transfection mixtures were incubated at 37°C for 12 h. The cells were infected at an MOI of 1 with each miRT-CVA21 for 2 h at 37°C in serum-free media. Following infection, the media and unincorporated virus were removed and replaced with 100 μL complete growth media, and the cells were incubated at 37°C in 5% CO_2. Twenty-four hours postinfection, the supernatants were collected and titrated as described above. The cells were assayed for proliferation using a 3-(4,5-dimethylthiazolyl-2)-2,5-diphenyltetrazolium bromide (MTT) kit (30-1010K; ATCC).

Genetic Stability of miRNA Targets

TE671 cells were grown in medium supplemented with 2% horse serum for 4 days (dTE671). dTE671 cells were initially infected with miRNA-detargeted CVA21 at an MOI of 10, and samples were collected 24 h later. All samples were subjected to three freeze-thaw cycles, and the lysates were clarified by centrifugation and filtered through a 0.22-μm filter. Virus in clarified lysates was passaged serially in dTE671 cells, each time using 1 vol clarified lysate to 2 vol fresh media. Viral RNA was isolated from the cleared lysates with a QIAamp viral RNA mini kit (52906; QIAGEN). cDNA was synthesized using Superscript III first-strand synthesis system (18080-051; Life Technologies; Grand Island, NY, USA). Regions containing the response elements were amplified using Platinum *Taq* DNA polymerase kit (10966-034; Thermo Fisher Scientific) and amplicons bulk sequenced.

Animal Experiments

The Mayo Clinic Institutional Animal Care and Use Committee approved all animal studies. Five- to six-week-old female CB17 ICR-SCID (Institute of Cancer Research-Severe Combined Immunodeficiency) mice were purchased from Envigo (Huntington, Cambridgeshire, UK). The mice were irradiated with 150 cGy to suppress the innate immune system and allow consistent tumor implantation. At 24 h postirradiation, mice were implanted subcutaneously with 5×10^6 Mel624 cells in the right flank. When tumors reached an average of 0.5 cm × 0.5 cm, the tumors were injected with 1–32 μg *in vitro*-derived RNA in 50 μL saline. All tumor-bearing mice were observed daily and mice weighed, and tumor size was measured using a handheld caliper. At the time of euthanasia, mice were anesthetized through the inhalation of isoflurane, and blood was obtained through cardiac puncture. Tissues were harvested, immediately sectioned, and flash frozen for virus titration and response element genetic stability analysis. Notably, tumor volume and weight data for control- and

CVA21-treated cohorts in Figures 1 and 4 are repeat graphs because all miRNA-detargeted constructs were tested simultaneously in a single experiment.

Serum Virus Titration

Mice were anesthetized through the inhalation of isoflurane, and blood was collected from the submandibular vein in a BD Microtainer tube (365967; BD Biosciences; San Jose, CA, USA). Blood was allowed to coagulate for 30 min at room temperature followed by serum separation by centrifugation at 8,000 rpm for 5 min. Viral loads were determined by titrating on H1-HeLa cells as described above.

Response Element Stability in Tissues

Total RNA was isolated from frozen tissue sections using an RNeasy plus universal mini kit (73404; QIAGEN) according to the manufacturer's instructions. cDNA was synthesized using Superscript III first-strand synthesis system (18080-051; Life Technologies). Regions containing the response elements were amplified using Platinum *Taq* DNA polymerase kit (10966-034; Thermo Fisher Scientific) and amplicons bulk sequenced.

RNA Modeling

Secondary RNA structures were generated using the IPknot web server (http://rtips.dna.bio.keio.ac.jp/ipknot/) available through the Graduate School of Information Science, Nara Institute of Science and Technology Japan. 3′ NCR predictions were predicted using level 3 (pseudoknotted with nested pseudoknots) prediction, CONTRAfold scoring model, with refinements. 5′ NCR predictions were predicted using level 2 (nested pseudoknots), CONTRAfold scoring model, with refinements.

Statistical Analysis

GraphPad Prism software, version 7c (GraphPad Software), was used for data analysis and graphical representations. Survival curves were plotted according to the Kaplan-Meier method, and the survival rates across treatment groups were compared using log rank tests with 95% confidence interval. A sample size of five animals per group was used because it will have at least 80% power to detect a minimum difference of two standard deviations between group measurements. In order to account for multiple comparison issues, the power of the experiments assumes a type I error with an alpha = 0.05. Two-sided nonparametric Steel multiple comparisons with mock infection controls were used for statistical analysis of miRNA mimic targeting assays, and $p < 0$ 0.05 was considered statistically significant. Mean comparisons using Dunnett's method with a CVA21 infection control were used for statistical analysis of cytotoxicity in primary human skeletal muscle cells, and $p < 0.05$ was considered statistically significant. MP Pro 13 software (SAS Institute) was used for determining significance. Test results for the miRNA mimic targeting assays are shown in Table S1.

Data Availability

Data are available from the corresponding author upon reasonable request.

SUPPLEMENTAL INFORMATION

Supplemental Information can be found online at https://doi.org/10.1016/j.omto.2020.05.003.

AUTHOR CONTRIBUTIONS

N.B.E., S.J.R., and A.J.S. conceived the project. N.B.E. and A.J.S. performed *in vitro* experiments. R.A.N. and A.J.S. performed *in vivo* experiments. A.J.S. performed the RNA predictions. N.B.E., S.J.R., and A.J.S. wrote the manuscript. S.J.R. and A.J.S. supervised the project.

CONFLICTS OF INTEREST

S.J.R. is the CEO of Vyriad, which holds a patent for "treating cancer with viral nucleic acid." The authors have filed a patent application regarding the technology presented in this paper.

ACKNOWLEDGMENTS

We thank E. Rieder and E. Wimmer (State University of New York-Stony Brook) for the initial construction of an infectious CVA21 clone. Additionally, we thank A. Dufresne and M. Gromeier (Duke University) for kindly providing the CVA21 clone. We thank Dr. Jim Maher III (Mayo Clinic) for communications regarding RNA structural analysis. We thank the Mayo Clinic Center for Clinical and Translational Sciences biostatisticians for their help with statistical analyses. We also sincerely and respectfully acknowledge the revolutionary contribution to medical research made possible by Henrietta Lacks and HeLa cells. The graphical abstract was created with BioRender.com. This work was funded by Al and Mary Agnes McQuinn and Mayo Clinic, United States.

REFERENCES

1. Andtbacka, R.H., Curti, B., Kaufman, H., Daniels, G.A., Spitler, L.E., Lutzky, J., Hallmeyer, S., Whitman, E.D., Nemunaitis, J.J., Schultz, S., et al. (2015). Final data from CALM: a phase II study of Coxsackievirus A21 (CVA21) oncolytic virus immunotherapy in patients with advanced melanoma. https://viralytics.com/wp-content/uploads/2015/03/150601-AmSocClinOnc-CALM-Poster.pdf.
2. Pandha, H.S., Ralph, C., Harrington, K.J., Mansfield, D., Melcher, A., Feng, Z., Paustian, C., Bifulco, C., Fox, B., Quah, M.Y., et al. (2015). Phase I STORM study: intravenous delivery of a novel oncolytic immunotherapy agent, CAVATAK, in advanced cancer patients. https://viralytics.com/wp-content/uploads/2015/11/2015-SITC-Final-STORM-poster.pdf.
3. Pandha, H., Harrington, K., Ralph, C., Melcher, A., and Shafren, D.R. (2015). Intravenous delivery of a novel oncolytic immunotherapy agent, CAVATAK, in advanced cancer patients. Cancer Res. *75* (*Suppl 15*), CT205.
4. Pandha, H.S., Annels, N., Simpson, G., Michael, A., Mansfield, D., Sandhu, S.S., Melcher, A.A., Harrington, K.J., Davies, B., Au, G., et al. (2017). Phase I/II CANON study: oncolytic immunotherapy for the treatment of non-muscle invasive bladder cancer using intravesical coxsackievirus A21. https://viralytics.com/wp-content/uploads/2017/03/Final-CANON-EACR-AACR-SIC-2017.pdf.
5. Pandha, H.S. (2018). Clinical evaluation of a novel oncolytic immunotherapy agent, CAVATAK® in combination with immune checkpoint therapy in advanced cancer patients. https://www.viralytics.com/our-pipeline/scientific-presentations/scientific-presentations-2018/.
6. Dekel, B., Yoeli, R., Shulman, L., Padeh, S., and Passwell, J.H. (2002). Localized thigh swelling mimicking a neoplastic process: involvement of coxsackie virus type A21. Acta Paediatr. *91*, 357–359.
7. Kelly, E.J., Hadac, E.M., Greiner, S., and Russell, S.J. (2008). Engineering microRNA responsiveness to decrease virus pathogenicity. Nat. Med. *14*, 1278–1283.

8. Carlisle, R.C., Briggs, S.S., Hale, A.B., Green, N.K., Fisher, K.D., Etrych, T., Ulbrich, K., Mautner, V., and Seymour, L.W. (2006). Use of synthetic vectors for neutralising antibody resistant delivery of replicating adenovirus DNA. Gene Ther. *13*, 1579–1586.
9. Li, S., and Ma, Z. (2001). Nonviral gene therapy. Curr. Gene Ther. *1*, 201–226.
10. Ohlfest, J.R., Freese, A.B., and Largaespada, D.A. (2005). Nonviral vectors for cancer gene therapy: prospects for integrating vectors and combination therapies. Curr. Gene Ther. *5*, 629–641.
11. Al-Dosari, M.S., and Gao, X. (2009). Nonviral gene delivery: principle, limitations, and recent progress. AAPS J. *11*, 671–681.
12. Pascolo, S. (2017). Messenger RNA: The Inexpensive Biopharmaceutical. J. Multidiscip. Eng. Sci. Technol. *4*, 6937–6941.
13. Hadac, E.M., Kelly, E.J., and Russell, S.J. (2011). Myeloma xenograft destruction by a nonviral vector delivering oncolytic infectious nucleic acid. Mol. Ther. *19*, 1041–1047.
14. Barnes, D., Kunitomi, M., Vignuzzi, M., Saksela, K., and Andino, R. (2008). Harnessing endogenous miRNAs to control virus tissue tropism as a strategy for developing attenuated virus vaccines. Cell Host Microbe *4*, 239–248.
15. Kelly, E.J., and Russell, S.J. (2009). MicroRNAs and the regulation of vector tropism. Mol. Ther. *17*, 409–416.
16. Ruiz, A.J., and Russell, S.J. (2015). MicroRNAs and oncolytic viruses. Curr. Opin. Virol. *13*, 40–48.
17. Ruiz, A.J., Hadac, E.M., Nace, R.A., and Russell, S.J. (2016). MicroRNA-Detargeted Mengovirus for Oncolytic Virotherapy. J. Virol. *90*, 4078–4092.
18. Boyer, J.C., and Haenni, A.L. (1994). Infectious transcripts and cDNA clones of RNA viruses. Virology *198*, 415–426.
19. Pilipenko, E.V., Maslova, S.V., Sinyakov, A.N., and Agol, V.I. (1992). Towards identification of cis-acting elements involved in the replication of enterovirus and rhinovirus RNAs: a proposal for the existence of tRNA-like terminal structures. Nucleic Acids Res. *20*, 1739–1745.
20. Pilipenko, E.V., Poperechny, K.V., Maslova, S.V., Melchers, W.J., Slot, H.J., and Agol, V.I. (1996). Cis-element, oriR, involved in the initiation of (-) strand poliovirus RNA: a quasi-globular multi-domain RNA structure maintained by tertiary ('kissing') interactions. EMBO J. *15*, 5428–5436.
21. Melchers, W.J., Hoenderop, J.G., Bruins Slot, H.J., Pleij, C.W., Pilipenko, E.V., Agol, V.I., and Galama, J.M. (1997). Kissing of the two predominant hairpin loops in the coxsackie B virus 3′ untranslated region is the essential structural feature of the origin of replication required for negative-strand RNA synthesis. J. Virol. *71*, 686–696.
22. Mirmomeni, M.H., Hughes, P.J., and Stanway, G. (1997). An RNA tertiary structure in the 3′ untranslated region of enteroviruses is necessary for efficient replication. J. Virol. *71*, 2363–2370.
23. Wang, J., Bakkers, J.M., Galama, J.M., Bruins Slot, H.J., Pilipenko, E.V., Agol, V.I., and Melchers, W.J. (1999). Structural requirements of the higher order RNA kissing element in the enteroviral 3'UTR. Nucleic Acids Res. *27*, 485–490.
24. van Ooij, M.J., Glaudemans, D.H., Heus, H.A., van Kuppeveld, F.J., and Melchers, W.J. (2006). Structural and functional integrity of the coxsackievirus B3 oriR: spacing between coaxial RNA helices. J. Gen. Virol. *87*, 689–695.
25. Zoll, J., Heus, H.A., van Kuppeveld, F.J., and Melchers, W.J. (2009). The structure-function relationship of the enterovirus 3′-UTR. Virus Res. *139*, 209–216.
26. Binder, J.J., Hoffman, M.A., and Palmenberg, A.C. (2003). Genetic stability of attenuated mengovirus vectors with duplicate primary cleavage sequences. Virology *312*, 481–494.
27. Rivera, V.M., Welsh, J.D., and Maizel, J.V., Jr. (1988). Comparative sequence analysis of the 5′ noncoding region of the enteroviruses and rhinoviruses. Virology *165*, 42–50.
28. Pilipenko, E.V., Blinov, V.M., Romanova, L.I., Sinyakov, A.N., Maslova, S.V., and Agol, V.I. (1989). Conserved structural domains in the 5′-untranslated region of picornaviral genomes: an analysis of the segment controlling translation and neurovirulence. Virology *168*, 201–209.
29. Skinner, M.A., Racaniello, V.R., Dunn, G., Cooper, J., Minor, P.D., and Almond, J.W. (1989). New model for the secondary structure of the 5′ non-coding RNA of poliovirus is supported by biochemical and genetic data that also show that RNA secondary structure is important in neurovirulence. J. Mol. Biol. *207*, 379–392.
30. Andino, R., Rieckhof, G.E., and Baltimore, D. (1990). A functional ribonucleoprotein complex forms around the 5′ end of poliovirus RNA. Cell *63*, 369–380.
31. Andino, R., Rieckhof, G.E., Achacoso, P.L., and Baltimore, D. (1993). Poliovirus RNA synthesis utilizes an RNP complex formed around the 5′-end of viral RNA. EMBO J. *12*, 3587–3598.
32. Barton, D.J., O'Donnell, B.J., and Flanegan, J.B. (2001). 5′ cloverleaf in poliovirus RNA is a cis-acting replication element required for negative-strand synthesis. EMBO J. *20*, 1439–1448.
33. Vogt, D.A., and Andino, R. (2010). An RNA element at the 5′-end of the poliovirus genome functions as a general promoter for RNA synthesis. PLoS Pathog. *6*, e1000936.
34. Burrill, C.P., Westesson, O., Schulte, M.B., Strings, V.R., Segal, M., and Andino, R. (2013). Global RNA structure analysis of poliovirus identifies a conserved RNA structure involved in viral replication and infectivity. J. Virol. *87*, 11670–11683.
35. Slobodskaya, O.R., Gmyl, A.P., Maslova, S.V., Tolskaya, E.A., Viktorova, E.G., and Agol, V.I. (1996). Poliovirus neurovirulence correlates with the presence of a cryptic AUG upstream of the initiator codon. Virology *221*, 141–150.
36. Huang, P.N., Lin, J.Y., Locker, N., Kung, Y.A., Hung, C.T., Lin, J.Y., Huang, H.I., Li, M.L., and Shih, S.R. (2011). Far upstream element binding protein 1 binds the internal ribosomal entry site of enterovirus 71 and enhances viral translation and viral growth. Nucleic Acids Res. *39*, 9633–9648.
37. Liauw, W., Chern, B., and Shafren, D.R. (2012). Phase I, Open-Label, Cohort Study of CAVATAK (Coxsackievirus A21) Given Intravenously to Stage IV Patients Bearing ICAM-1 Expressing Solid Tumours. Eur. J. Cancer *48*, 180–181.
38. Andtbacka, R.H., Shafren, D.R., Grose, M., Post, L., and Weisberg, J. (2014). CAVATAK-mediated oncolytic immunotherapy in advanced melanoma patients. Cancer Res. *74* (*Suppl 19*), 2939.
39. Simpson, G.R., Annels, N., Ajaz, M., Launchbury, F.A., Bolton, G., Melcher, A.A., Harrington, K.J., Au, G.G., Shafren, D.R., and Pandha, H. (2014). Major synergy between Coxsackievirus A21 (CAVATAK (TM)) and radiotherapy or chemotherapy in bladder cancer. Eur. J. Cancer *50*, 41–42.
40. Dyer, A., Baugh, R., Chia, S.L., Frost, S., Iris, Jacobus, E.J., Khalique, H., Pokrovska, T.D., Scott, E.M., Taverner, W.K., et al. (2019). Turning cold tumours hot: oncolytic virotherapy gets up close and personal with other therapeutics at the 11th Oncolytic Virus Conference. Cancer Gene Ther. *26*, 59–73.
41. Shafren, D.R., Dorahy, D.J., Greive, S.J., Burns, G.F., and Barry, R.D. (1997). Mouse cells expressing human intercellular adhesion molecule-1 are susceptible to infection by coxsackievirus A21. J. Virol. *71*, 785–789.
42. Shafren, D.R., Dorahy, D.J., Ingham, R.A., Burns, G.F., and Barry, R.D. (1997). Coxsackievirus A21 binds to decay-accelerating factor but requires intercellular adhesion molecule 1 for cell entry. J. Virol. *71*, 4736–4743.
43. Johansson, E.S., Xing, L., Cheng, R.H., and Shafren, D.R. (2004). Enhanced cellular receptor usage by a bioselected variant of coxsackievirus a21. J. Virol. *78*, 12603–12612.
44. Newcombe, N.G., Beagley, L.G., Christiansen, D., Loveland, B.E., Johansson, E.S., Beagley, K.W., Barry, R.D., and Shafren, D.R. (2004). Novel role for decay-accelerating factor in coxsackievirus A21-mediated cell infectivity. J. Virol. *78*, 12677–12682.
45. Annels, N.E., Arif, M., Simpson, G.R., Denyer, M., Moller-Levet, C., Mansfield, D., Butler, R., Shafren, D., Au, G., Knowles, M., et al. (2018). Oncolytic Immunotherapy for Bladder Cancer Using Coxsackie A21 Virus. Mol. Ther. Oncolytics *9*, 1–12.

OMTO, Volume *17*

Supplemental Information

Oncolytic Activity of Targeted Picornaviruses Formulated as Synthetic Infectious RNA

Noura B. Elsedawy, Rebecca A. Nace, Stephen J. Russell, and Autumn J. Schulze

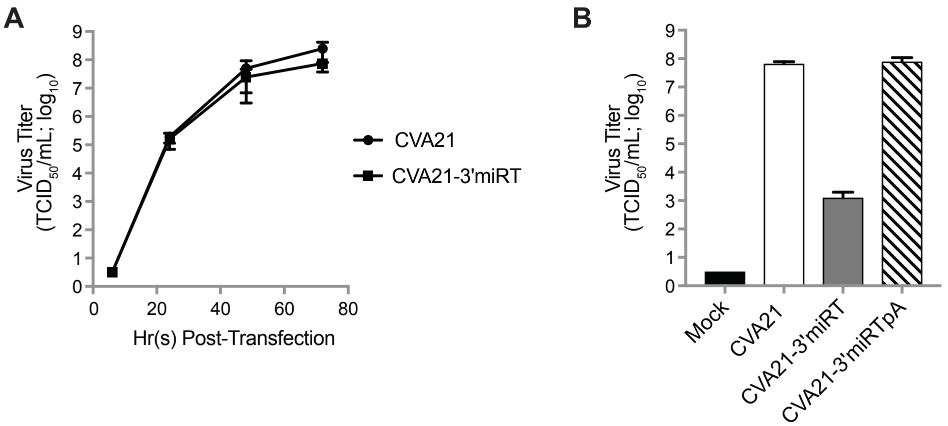

Figure S1. CVA21-3'miRT iRNA specific infectivity is temperature-sensitive and is improved with *in vitro* polyadenylation. (**A**) Infectious virus recovery time course in H1-HeLa cells at 32 °C. Cells were transfected with 1 μg of *in vitro*-derived RNA encoding CVA21 or CVA21-3'miRT genomes. Samples were collected at various times post transfection and infectious virus titrated. The experiment was run in quadruplicate and the data is represented as mean viral titers +/- standard error. (**B**) H1-HeLa cells were transfected with 1 μg of *in vitro*-derived RNA encoding CVA21, CVA21-3'miRT, or polyadenylated CVA21-3'miRT (pA). Recovered viruses were passaged twice in H1-HeLa cells and the cleared stocks titrated. The experiment was run in duplicate and that data are represented as mean viral titer +/- standard deviation.

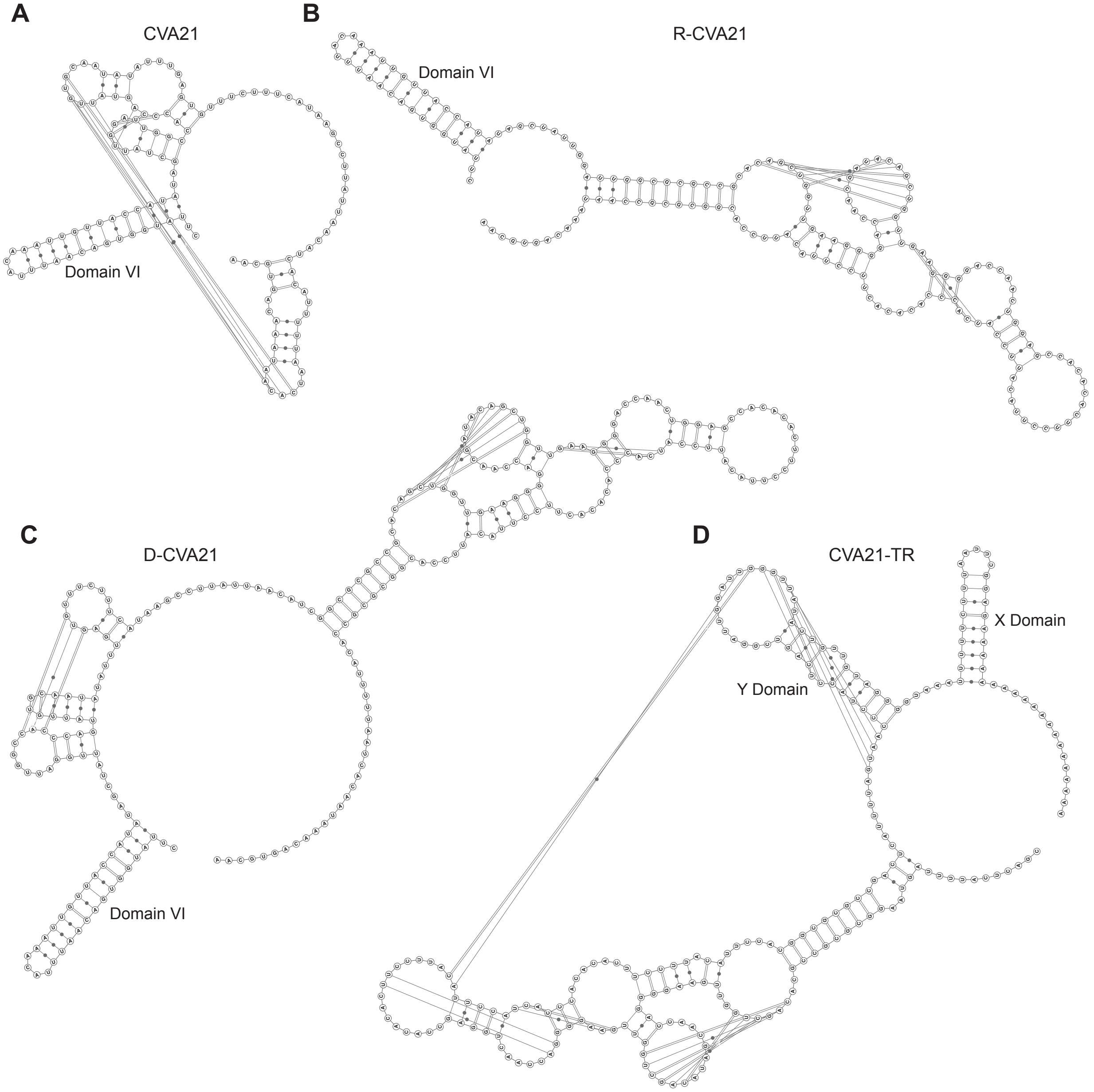

Figure S2. Predicted secondary RNA structures of microRNA-detargeted CVA21 NCRs. Predicted pseudoknot interactions are depicted by lines. Predictions of structures of the regions spanning domain VI and the downstream spacer in the 5' NCR for CVA21 (**A**), R-CVA21 (**B**), D-CVA21 (**C**), or the 3' NCR of CVA21-TR (**D**).

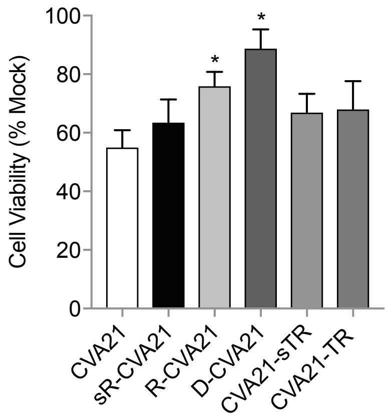

Figure S3. *In vitro* characterization of microRNA-detargeting efficacy in differentiated primary human skeletal muscle cells. Primary human skeletal muscle cells were incubated in differentiation media for 96 hrs. Differentiated cells were infected with unmodified or miRT-CVA21 at an MOI of 1. Cell viability was measured at 48 hrs post infection. This experiment was repeated in triplicate and the data are represented as mean cell viability +/- standard deviations. *$p < 0.05$ compared to CVA21 infection was considered significant.

Virus	X	Y	Level	-Level	Score Mean Diff	Std Err Diff	Z	p-Value	Hodges-Lehmann	Lower CL	Upper CL	Difference Plot	Median	Significant
CVA21	Condition	% Alive	miR142	Mock Infection	1.6	1.914854	0.835573	0.8191	1.36209	-3.98812	8.547545	1.3620885	3.156059	N
CVA21	Condition	% Alive	miR-133	Mock Infection	0.4	1.914854	0.208893	0.9987	0.30378	-4.43435	5.277986	0.3037818	4.858669	N
CVA21	Condition	% Alive	miR-206	Mock Infection	-0.4	1.914854	-0.20889	0.9987	-0.11628	-4.74996	5.307162	-0.1162759	4.96664	N
CVA21	Condition	% Alive	Combination	Mock Infection	0.4	1.914854	0.208893	0.9987	1.20778	-4.12101	6.503142	1.2077765	3.338805	N
sR-CVA21	Condition	% Alive	miR142	Mock Infection	2	1.914854	1.044466	0.6792	1.66108	-7.13336	10.09741	1.6610836	3.455054	N
sR-CVA21	Condition	% Alive	miR-133	Mock Infection	-1.6	1.914854	-0.83557	0.8191	-0.93815	-18.0799	8.408737	-0.9381508	5.747349	N
sR-CVA21	Condition	% Alive	miR-206	Mock Infection	-4.8	1.914854	-2.50672	0.0421	-42.10847	-109.464	-22.8808	-42.108469	47.22461	Y
sR-CVA21	Condition	% Alive	Combination	Mock Infection	-4.8	1.914854	-2.50672	0.0421	-67.01642	-87.9534	-44.3064	-67.016417	72.13255	Y
R-CVA21	Condition	% Alive	miR142	Mock Infection	0	1.914854	0	1	0.34846	-3.83397	6.130802	0.3484557	4.102876	N
R-CVA21	Condition	% Alive	miR-133	Mock Infection	-4.8	1.914854	-2.50672	0.0421	-15.84674	-38.4094	-5.07339	-15.846737	18.7038	Y
R-CVA21	Condition	% Alive	miR-206	Mock Infection	-4.8	1.914854	-2.50672	0.0421	-92.19014	-101.639	-41.3396	-92.190139	95.0472	Y
R-CVA21	Condition	% Alive	Combination	Mock Infection	-4.8	1.914854	-2.50672	0.0421	-67.40677	-73.0015	-16.2039	-67.406772	70.47463	Y
D-CVA21	Condition	% Alive	miR142	Mock Infection	0	1.914854	0	1	0.29485	-5.22073	8.960566	0.2948469	4.302206	N
D-CVA21	Condition	% Alive	miR-133	Mock Infection	-3.6	1.914854	-1.88004	0.1838	-35.98738	-61.8388	3.144034	-35.987376	40.23144	N
D-CVA21	Condition	% Alive	miR-206	Mock Infection	-4.8	1.914854	-2.50672	0.0421	-106.95717	-120.608	-66.4003	-106.95717	110.4039	Y
D-CVA21	Condition	% Alive	Combination	Mock Infection	-4.8	1.914854	-2.50672	0.0421	-62.56788	-86.0686	-53.9022	-62.567882	63.65272	Y
CVA21-sTR	Condition	% Alive	miR142	Mock Infection	0.4	1.914854	0.208893	0.9987	0.44849	-3.43769	9.98751	0.4484926	3.372	N
CVA21-sTR	Condition	% Alive	miR-133	Mock Infection	-3.2	1.914854	-1.67115	0.2743	-5.56374	-15.4224	3.986601	-5.5637443	8.27973	N
CVA21-sTR	Condition	% Alive	miR-206	Mock Infection	-4.8	1.914854	-2.50672	0.0421	-44.88248	-55.5044	-11.4917	-44.882478	55.82902	Y
CVA21-sTR	Condition	% Alive	Combination	Mock Infection	-4	1.914854	-2.08893	0.1177	-49.29266	-60.8636	0.041527	-49.292655	54.64134	N
CVA21-TR	Condition	% Alive	miR142	Mock Infection	1.2	1.914854	0.62668	0.9246	1.12578	-3.65968	7.313814	1.1257801	3.629468	N
CVA21-TR	Condition	% Alive	miR-133	Mock Infection	-2.4	1.914854	-1.25336	0.5295	-3.45313	-10.1213	3.006561	-3.4531284	6.96632	N
CVA21-TR	Condition	% Alive	miR-206	Mock Infection	-2.4	1.914854	-1.25336	0.5295	-3.81484	-25.0441	3.347083	-3.8148401	4.983251	N
CVA21-TR	Condition	% Alive	Combination	Mock Infection	-2	1.914854	-1.04447	0.6792	-3.23346	-63.1956	3.928463	-3.2334609	5.423438	N

Table S1. Nonparametric Steel multiple comparisons with mock infection controls statistical analysis of microRNA targeting efficiency of samples described in Fig. 3A.
